# RETRACTION: Comparative Study on Gene Expression in Psoriatic Lesions Versus Chronic Wound Healing Processes

**DOI:** 10.1111/iwj.70487

**Published:** 2025-04-02

## Abstract

**RETRACTION:** QiongZ. , DongxueF. , QingZ. , YukunR. , and YuepengA. , “Comparative Study on Gene Expression in Psoriatic Lesions Versus Chronic Wound Healing Processes,” International Wound Journal 21, no. 3 (2024): e14532, 10.1111/iwj.14532.38012097 PMC10898408

The above article, published online on 27 November 2023, in Wiley Online Library (http://onlinelibrary.wiley.com/), has been retracted by agreement between the journal Editor in Chief, Professor Keith Harding; and John Wiley & Sons Ltd. Following an investigation by the publisher, all parties have concluded that this article was accepted solely on the basis of a compromised peer review process. The editors have therefore decided to retract the article. The authors did not respond to our notice regarding the retraction.

**RETRACTION:** Z. Qiong , F. Dongxue , Z. Qing , R. Yukun , and A. Yuepeng , “Comparative Study on Gene Expression in Psoriatic Lesions Versus Chronic Wound Healing Processes,” International Wound Journal 21, no. 3 (2024): e14532, 10.1111/iwj.14532.38012097 PMC10898408

The above article, published online on 27 November 2023, in Wiley Online Library (http://onlinelibrary.wiley.com/), has been retracted by agreement between the journal Editor in Chief, Professor Keith Harding; and John Wiley & Sons Ltd. Following an investigation by the publisher, all parties have concluded that this article was accepted solely on the basis of a compromised peer review process. The editors have therefore decided to retract the article. The authors did not respond to our notice regarding the retraction.

